# Psychological distress and smoking behaviors of Chinese college students: mediating effects of the dimensions of learning burnout

**DOI:** 10.1186/s40359-022-00840-6

**Published:** 2022-05-19

**Authors:** Xiong Li, Yuhua Tan, Shanqing Li, Xiaoxin Wang

**Affiliations:** 1 Honghe Health Vocational College, Mengzi City, Honghe Prefecture, Yunnan Province China; 2 Yunnan Technology and Business University, Kunming City, Yunnan Province China; 3 Honghe Vocational and Technical College, Mengzi City, Honghe Prefecture, Yunnan Province China

**Keywords:** Psychological distress, Learning burnout, Smoking behavior, The theory of planned behavior

## Abstract

**Objectives:**

Smokers or never smokers exposed to environmental tobacco use are usually associated with various diseases and cancers. In order to better help college students prevent the tobacco use and thus lower the incidence of avoidable diseases, this study explored the predictive power of different variables including demographic and psychological variables in relation to smoking behaviors.

**Methods:**

Maslach Burnout Inventory-Student Survey and Kessler Psychological Distress Scale (K10) were used in this study.

**Results:**

There were 1449 college students participating in the study with 1340 pieces of valid data left, the effective ratio was 92.48%. The valid data included 37.1% male and 62.9% female aged 18.83 on average with 1.55 standard deviation. The multivariate logistic regression indicated that college students who were male (versus female, OR = 9.55), majoring in medicine and sports (versus nursing, OR_medicine_ = 2.19, OR_sports_ = 2.81), born in the non-singleton family (versus singleton family, OR = 0.63) with higher family income (versus lower family income, OR = 0.45), surrounded with smoking friends (versus without smoking friends, OR = 0.18), were more vulnerable to smoke. In addition, combined with the theory of planned behavior, the dimensions of learning burnout had full mediation effects between psychological distress and smoking behavior.

**Conclusions:**

Psychological distress can only indirectly affect smoking behavior via learning efficacy, cynicism and emotional exhaustion. Adjustments from different dimensions of learning burnout such as avoiding cynicism about learning, enhancing learning efficacy and emotion exhaustion will help college students better prevent the tobacco use.

## Introduction

Tobacco consumption is usually considered to be a double-edged sword which has negative impacts on public health and human longevity regardless of its positive effect on economic growth. Studies have found that as an unhealthy lifestyle, cigarette smoking could reduce people’s life span by an average of 7 years due to the complex compounds that cigarettes may contain, while most of which are carcinogens in human [1]. Those who got used to smoke in public not only undermined their own physical health, but also had the health of people around them endangered when they were smoking.

Cigarette smoking is presently found to be positively associated with nearly 40 diseases and causes of death for human, such as vascular diseases, oral diseases, chronic obstructive pulmonary disease (COPD), cancer of the lung, cancer of the liver, cancer of pancreas, cancer of renal pelvis and so on [2]. Smokers who have already formed the smoking habits are more likely to suffer from lung cancers than never smokers [3]. However, never smokers who expose themselves to environmental tobacco smoke (ETS) are also at high risks of various chronic diseases and cancers [4].

China is the country with the biggest tobacco consumption in the world [5]. The number of smokers in China had reached at least three hundred million in 2011 [6]. It was reported that the overall prevalence of tobacco smoking in China was up to 31.4% accounting for a quarter of the world’s total smokers, while the total premature mortality resulting from cigarette smoking was 7.9% and the tobacco-related deaths were estimated to be 1 million annually in China [7, 8]. These reported data may suggest that the tobacco control in China still needs improvement.

Generally speaking, the more the tobacco is consumed and the earlier the individual smokes, the greater the harm to the body as well as the higher the addiction will be. In terms of the incidence of different cancers in China, the incidence of lung cancer ranked fourth in the 1970s and 1990s, whereas the incidence of lung cancer was constantly upgrading and had reached the highest among all kinds of cancers since 2000, the death rate from lung cancer had increased by 465% in the last 30 years accompanying with the remarkable increase of tobacco consumption [9].

The relation between physical health and cigarette smoking has been revealed in many studies, these studies have also indicated the current situation of smoking control in China is not optimistic, especially for Chinese youths and college students [10, 11]. College students in China are more likely to smoke than others for some reasons. Firstly, Chinese culture is featured by Confucian and collectivism [12]. Given this kind of cultural influence, college students commonly prefer to imitate peers’ smoking behaviors in order to socially integrate into the surroundings. Smoking has become the social label and peer bond for their relationships with others. Moran [13] has identified these young smokers as “social smokers” who show less intentions to quit smoking; secondly, college students are usually interested in the things they have never come across. The curiosity on the new things and new experiences may lead them easily to smoke; thirdly, college students in China have to face fierce competition and learning stress at school. The competition and learning stress can increase the risks of smoking through which college students enable themselves to control and relieve their feelings of tension and stress [14].

In most cases, cigarette smoking is usually viewed as the method to mitigate one’s anxiety and depression. For example, Hong et al. [15] applied different questionnaires to investigate 1068 fishermen, the impact of nicotine dependence upon negative emotions was revealed that nicotine dependence could partially counteract the negative effect of work stress upon depression and anxiety. With heavy work stress, fishermen often exhibited high levels of depression and anxiety. By the inhaling of cigarettes, the levels of depression and anxiety could be reduced for fishermen. Picciotto et al. [16] had also argued that the mitigation of negative emotions through cigarette smoking existed from the perspective of physiology, it was concluded that the physiological mechanism of cigarette smoking was due to the broad expression of nicotinic acetylcholine receptors (nAChRs), which further ascertained the positive effect of tobacco use upon emotional adjustment.

Despite the positive effect of tobacco use through which one can alleviate the levels of depression and anxiety, tobacco use is still harmful to the body. Moreover, excessive nicotine intake always produces biological adaption that could lead to the opposite effect and strengthen the smokers’ existing depressions and anxieties, or even induce smoking-abstinence-related negative affect [16, 17]. Therefore, due to plenty of hazards of smoking behaviors, effective tobacco prevention programs conducted in China especially for Chinese college students are necessary.

### The present study

Although many studies have explored the impact of cigarette smoking upon emotion and cognition [15–18], few of them have discussed whether negative emotions could contribute to the enhanced susceptibility of smoking behavior or not. In other words, appropriate cigarette smoking will certainly help regulate negative emotions while the likelihood of emotion-induced smoking behavior is not yet unveiled.

In order to verify the conviction that negative emotions could facilitate college students’ smoking behaviors, learning burnout and psychological distress are taken into consideration in this article as the predictors of smoking behavior. Stoliker and Lafreniere [19] have pointed out that stressors such as learning burnout have become a common problem among students and something that they usually encounter during their educational career. Learning burnout is mainly the internal threat giving rise to college students’ mental health problems and unhealthy behavioral habits. Not only is learning burnout defined as a negative emotional state but also does it relate to psychological distress [19, 20]. The Chinese students who were haunted in the learning burnout usually failed the academic performance as well [21]. As a result, they would have to bear the enormous pressure from outside and inside, followed by the high levels of psychological distress and the increased inclinations of smoking.

However, due to a variety of factors including learning burnout and mental health problems might contribute to the inclination of cigarette smoking in theory, the influence of more possible variables would be explored in this article. This article aimed at building a multivariate logistic model to predict the smoking behaviors of college students based on a wide range of independent variables such as age, gender, majors, socioeconomic status (SES), family environment (FE), peer influence, learning burnout and psychological distress so that college students’ smoking behaviors could be accurately predicted and intervened. Among all these variables, learning burnout and psychological distress were expected to be strongly associated with smoking behaviors. Therefore, we will explore the relationships among learning burnout, psychological distress and smoking behavior through empirical research, discover the possible mediation mechanism behind these variables and figure out the predictive power of different variables on smoking behavior. For these purposes, we further made three assumptions as follows.

#### Hypothesis 1: Various demographic variables would predict smoking behavior

Previous literature had confirmed that smoking behavior was significantly associated with age, gender, majors, SES, FE, peer influence [22–24]. Those people featured with high age (versus low age), male (versus female), art majors (versus science and engineering majors), high SES (versus low SES), disharmonious and broken FE (versus harmonious and complete FE) were more likely to smoke. Thus, age, gender, majors, SES and FE would be the demographic variables included in this article to verify the findings of previous studies.

In addition, Wang et al. [24] had argued that friendship was also an inescapable factor that influence people’s smoking decisions. Based on the data they collected, Wang et al. [24] compared people who had the increased number of smoking friends from 1989 to 1993 with those who had the decreased number of smoking friends during the same period, they found that people with the increased number of smoking friends were more likely to smoke. It seems not only does the quality of the friendship matter, but also what kind of friends people usually choose to make may to some extent determine the smoking behaviors. Thus, for purpose of verifying the conviction that the type of friends college students chose to make would affect their smoking behaviors, the predictive power of peer influence on smoking behavior was evaluated by setting up an item like this: *do you have any friends who usually smoke around you*.

#### Hypothesis 2: Learning burnout and its different dimensions significantly affect smoking behavior

According to the theory of planned behavior (TPB) that was first come up with by Ajzen in 1988, perceived behavioral control (PBC), subjective norm (SN) and attitude, were constructed as the essential factors to predict individual’s specific intention and behavior [25]. PBC refers to people’s perception of the ease or difficulty of performing the behavior of interest. It was treated as the concept of self-efficacy which was first proposed by Bandura and his associates within a general framework [26]; Correlated with PBC in the TPB model, SN is a social factor that refers to the perceived social pressure to perform or not to perform the behavior, and attitude refers to the degree to which a person has a favorable or unfavorable appraisal of the behavior [26]. It was convinced that all of these constructs in TPB model were available to predict a variety of health behaviors in many specific contexts [27]. In term of smoking behavior, TPB had already been confirmed to be the effective model to predict smoking behavior with all of its contents including PBC, SN and attitude significantly related to smoking behavior [28].

Although TPB was useful to predict smoking behavior, the effect sizes between TPB constructs and smoking behavior were relatively small [29]. As Mohiyeddini et al. [30] have argued, while TPB commonly shows high predictive power with respect to intention, it often falls short in the prediction of behavior, the key role in bridging the intention-behavior gap is emotion. In this sense, emotion should be added as the antecedent variable of smoking behavior in the TPB model.

Following this clue, we assumed that learning burnout as an emotional factor might relate to smoking behavior closely. Ling et al. [31] have concluded that learning burnout has three dimensions: emotional exhaustion, cynicism and learning efficacy. Emotional exhaustion refers to students’ feelings of tiredness after learning. The individual who undergoes emotional exhaustion will excessively consume the inner cognitive resource and thus perform a sense of fatigue. He or she will eventually exhibit no extra enthusiasm and energy on learning and possibly other behaviors such as smoking. So theoretically, it was predicted that emotional exhaustion would lead to the decreased inclination of smoking behavior.

**Fig. 1:**
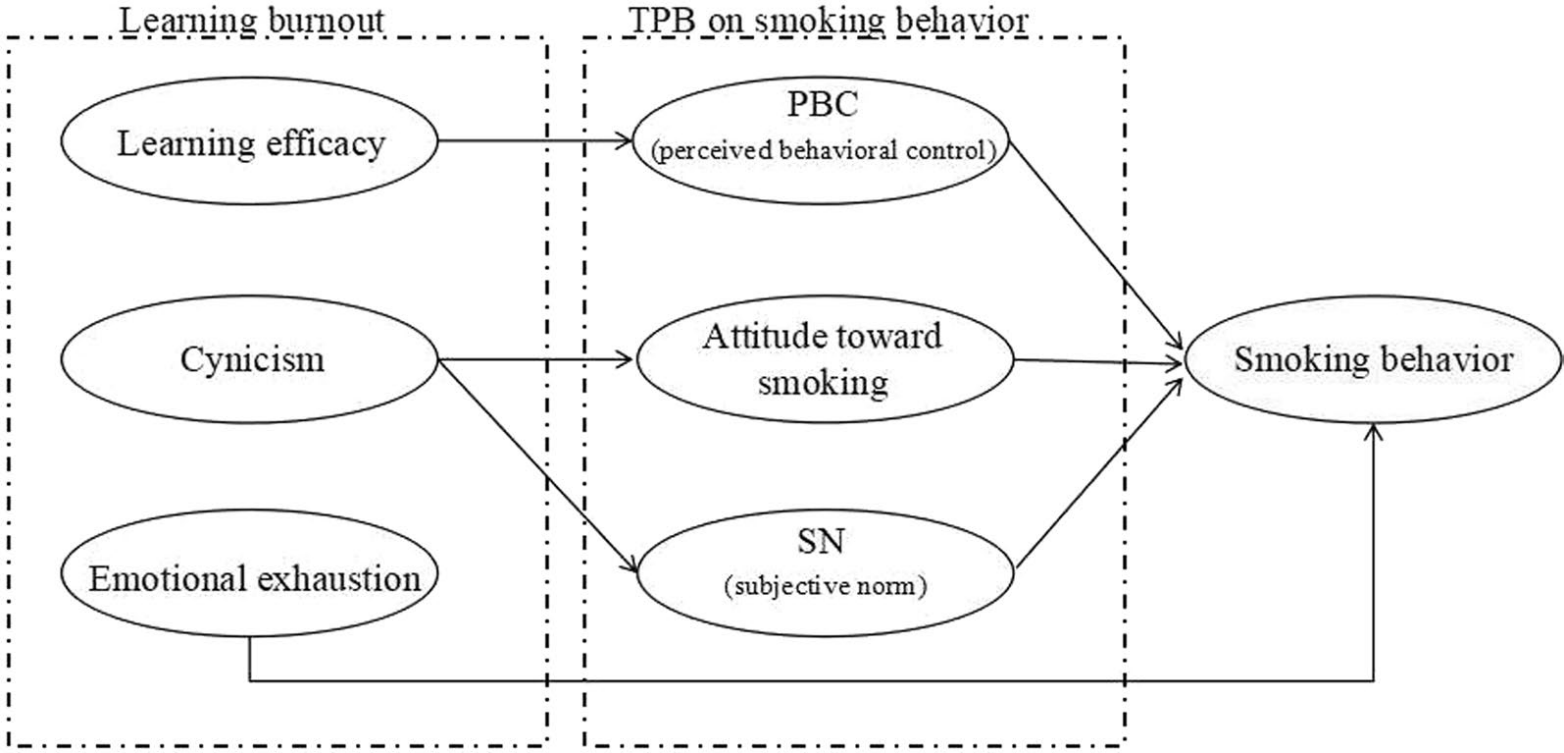
The relation between the dimensions of learning burnout and the constructs of TPB model toward smoking behavior

Besides the emotional exhaustion, the remaining dimensions of learning burnout are similar to the whole constructs in TPB model (as shown in Fig. 1). Since TPB model has been proven to be useful in the prediction of smoking behavior [29], it is thereby crucial to find out the theoretical connections between the dimensions of learning burnout and TPB model. However, these connections are made possible. Specifically, the three constructs in TPB model are found to correspond respectively to the two remaining dimensions of learning burnout.

As one of the dimensions of learning burnout, learning efficacy shares the common origin with PBC though their directions vary. That is, PBC in this case refers to the perceived ease or difficulty of smoking behavior management whereas learning efficacy points to the perceived ease or difficulty of learning. Due to the conceptual similarity between these two variables, we contended that learning efficacy could affect smoking behavior through PBC for people’s disposition of transferring similar knowledge from one area to another [32].

Cynicism, as another dimension of learning burnout, resembles the attitude and SN in TPB model. Cynicism by definition contains two meanings: one is that it represents the preference of learning or to what extent students hate to study, which can be regarded as the construct of attitude in TPB model; The other meaning that cynicism embodies is the students’ reflections on what kind of social influence learning would produce and what kind of social pressure they would have to face once the poor academic performances happen. Thus, SN which represents the perceived social pressure to quit smoking and cynicism which embodies social pressure to keep learning are the intertwined variables with conceptual similarity. From this point of view, cynicism can be regarded as SN or attitude toward learning behavior, with its highly probable influence on smoking behavior through SN and attitude toward smoking by transfer learning.

In sum, learning efficacy and cynicism are two dimensions of learning burnout corresponding to PBC, SN and attitude in TPB model. Meanwhile, according to the viewpoint that people’s knowledge gained from one specific case can then be transferred to the novel situation naturally [32], it is reasonable to predict that learning efficacy and cynicism can affect smoking behavior through PBC, SN and attitude. Consistent with the above-mentioned inferences, we hypothesize that learning burnout and all its different dimensions could affect smoking behavior.

#### Hypothesis 3: Learning burnout and its dimensions mediate the relation between psychological distress and smoking behavior

The self-medication hypothesis postulates that individuals always turn to smoking to alleviate the symptoms and therefore suggests that symptoms of depression and anxiety may lead to smoking [33]. Individuals with mental illness and symptoms of depression and anxiety tended to start smoking at an earlier age, smoke more heavily, and were more addicted to cigarettes than the general population [33]. Therefore, mental illness may play an important role on tobacco use, the link has often been taken to reflect a causal relationship, with poor mental health predisposing to regular tobacco use [34]. This phenomenon was specifically found in White individuals and pregnant women instead of Black or Hispanic individuals [28, 35].

Given the relation between mental health and smoking behavior, there is a plausible prospect that psychological distress which synthesizes all kinds of mental health problems is capable of affecting the occurrence of smoking behavior.

Although learning burnout and psychological distress are both likely to influence smoking behavior, in TPB model, learning burnout and all its dimensions are closer to smoking behavior than psychological distress. Likewise, some studies have shown that mental disorders can lead to lowered academic achievement, college dropout, and worse functioning in later life [36, 37], so we hypothesize psychological distress could affect the inclination of smoking behavior through learning burnout and its dimensions for the same purpose of revealing the concealed mechanism behind emotion-induced smoking behaviors for college students.

## Methods

### Participants

A cross sectional study was conducted. The questionnaires were administered to two colleges by convenient sampling. There was a total of 1449 college students participating in the study and the data were collected after completion by the college students. After carefully screening the missing or inconsistent responses from the collected data (e.g., the definition of inconsistent responses: the item 6: “*have you always smoked*” with students’ answers: “*no*” contradicted the item 7: “*how frequently have you smoked*” with the answers: “*i have smoked at least 100 times in a month*”), 1340 pieces of data were left for further analysis, the effective ratio was 92.48%. Correspondingly, the participants in the analyzed data were aged 18.83 on average with 1.55 standard deviation.

### Procedures

The study has been approved by the academic committees of Honghe Health Vocational college and Yunnan Technology and Business University since it was planned.The academic committee of Yunnan Technology and Business University approved the research protocol and conducted study oversight and monitoring. The academic committee of Honghe Health Vocational college signed the ethical approval after the scrutiny of the measuring tools and the design of study process.

Before measuring, online advertisements introducing the study were delivered through class groups. A sample of 1449 college students from different colleges volunteered to participate in the study with oral agreement and received two course credits as encouragement at the end of the study. The anonymity and purposes of the measurements were well known by the participants. All the participants gave informed consent to the measurements by checking the agreement boxes on the questionnaires and were required to be genuine to respond. Then the measurements were carried out at different times and places so as to strictly control the common method deviation [38]. The personal information of the participants (e.g., name, phone number, address) was not collected in this study for subjecting to the ethical standards.

### Measures

#### Learning burnout

Learning burnout was measured with Maslach Burnout Inventory-Student Survey (MBI-SS) [39]. This questionnaire stemmed from Maslach Burnout Inventory (MBI) which was originally used for the measurement of job burnout, previous studies found it not only applicable on workplace but also available for students, so MBI was revised and enriched to become the MBI-SS [40]. It was reported that the Cronbach’s alpha of MBI-SS was 0.84–0.90, test–retest reliability of MBI-SS was 0.67–0.89, concurrent validity for its various dimensions were obtained 0.74, 0.68 and 0.50 respectively [41].

In the present study, MBI-SS as a 7-points frequency rating scale comprised of 15 items with each item ranged from 0 (Never) to 6 (always) was used. MBI-SS has three dimensions such as emotional exhaustion, cynicism and learning efficacy, the Cronbach’s alpha for each of its dimensions in this study was 0.82, 0.82 and 0.85 respectively, the Cronbach’s alpha of the total scale was 0.86. Learning burnout was represented by the summation of each of its dimensions with reverse score of learning efficacy notified.

#### Psychological distress

The Kessler Psychological Distress Scale (K10) was constructed based on Item Response Theory (IRT) which was a validated 10-item scale measuring the non-specific symptom of psychological distress [42]. Each item in the K10 has 5 points ranged from 1 (None of the time) to 5 (Almost all of the time) representing the frequency rating of each item over the 4 weeks [43]. With higher score meaning worse mental health, the K10 in Chinese version was applied with its reliability and validity ever reported by Wen et al. [44] (e.g., Kappa is 0.70, split-half reliability is 0.71 and Cronbach’s alpha is 0.80). Based on this study, the internal consistency of the K10 in Chinese version was 0.93 which signified its reliability was acceptable in the study.

### Statistical analysis

Descriptive analysis, chi-square test and logistic regression model were adopted. The alpha value was set at 0.05. Data were analyzed using SPSS 18 (IBM Corp., Armonk, NY, United States).

Associations between demographic variables and smoking behavior were analyzed by chi-square test. Those demographic variables unrelated to smoking behavior, in this case which did not pass the chi-square test, would not enter into the logistic model on the next step. Subsequently, the set of independent variables in the multivariate logistic model would include the remaining demographic variables, psychological distress, learning burnout and all its dimensions whereas the dependent variable in the logistic model was occupied by the occurrence of smoking behavior. Finally, in order to investigate the mediating effects of learning burnout and its various dimensions, we use the mediation method proposed by Iacobucci [45] which is unlike the traditional mediation procedures because of the dichotomous dependent variable. Mediation method in this study complied with the following procedures:

1. Fit X and Y via a logistic model:
  $${\text{Y}} = e1 + cX.$$
  The intercept e1 and the slope *c* were produced along with their standard errors (SE).
2. Fit X and M via a linear regression model:
  $$M = e2 + aX.$$
  The slope *a* was indicated along with the corresponded SE. Collected the parameter estimate *a* and its standard error, *s*_*a*_.
3. Fit X, M and Y via a regression model:
  $$Y = e3 + c^{\prime}X + bM.$$
  Collected the parameter estimate *b* and its standard error, *s*_*b*_.
4. Using the collected parameter estimates and calculating the mediation effect by the following formula:
  $$Z_{Mediation} = \frac{{\frac{a}{{s_{a} }} \times \frac{b}{{s_{b} }}}}{{\sqrt {\left( {\frac{a}{{s_{a} }}} \right)^{2} + \left( {\frac{b}{{s_{b} }}} \right)^{2} + 1} }}.$$

Test *Z*_*Mediation*_ against a standard normal distribution, the mediation effect is found if *Z*_*Mediation*_ exceeds |1.96| for a 2-tailed test with *a* = 0.05 [42].

## Results

### Descriptive analysis and chi-square test

A demographic survey including different variables were practiced. Descriptive analysis and chi-square test were used to explore the performances of different variables upon smoking behavior.

As shown in Table 1, gender, majors, peer influence, SES and singleton family were significantly correlated with smoking behavior.

**Table 1:** Demographic characteristics of present study cohort of college students

Variables	Frequency (*n* = 1340) (%)	Smoking behaviour		
		Never smoke (*n* = 1037)	Have ever smoked (*n* = 303)	*χ*^2^
Gender				366.56**
Male	37.1	243 (48.89%)	254 (51.11%)	
Female	62.9	794 (93.86%)	49 (6.14%)	
Majors				201.24**
Pedagogy	29.2	356 (91.05%)	35 (8.95%)	
Medicine	23.9	243 (75.94%)	77 (24.06%)	
Literature and art	3.2	39 (90.70%)	4 (9.30%)	
Engineering	1.3	11 (61.11%)	7 (38.89%)	
Sports	23.4	159 (50.64%)	155 (49.36%)	
Nursing	19.0	229 (90.16%)	25 (9.84%)	
Ethnicity				10.45*
Han	55.8	580 (77.54%)	168 (22.46%)	
Yi	19.4	206 (79.23)	54 (20.77%)	
Hani	4.3	35 (60.34%)	23 (39.66%)	
Other minority	20.4	216 (78.83%)	58 (21.17%)	
Socioeconomic status (SES)				11.43**
< 3000 yuan	31.6	343 (81.09%)	80 (18.91%)	
3000–10,000 yuan	53.8	559 (77.53%)	162 (22.47%)	
> 10000 yuan	14.6	135 (68.88%)	61 (31.12%)	
Family environment (FE)				
Single-parent household	11.0	115 (77.70%)	33 (22.30%)	0.01
Two-parent household	89.0	922 (77.35%)	270 (22.65%)	
Singleton family	17.0	161 (70.61%)	67 (29.39%)	7.21**
Non-singleton family	83.0	876 (78.78%)	236 (21.22%)	
Domicile place				3.80
Countryside	82.6	868 (78.41%)	239 (21.59%)	
Town	17.4	169 (72.53%)	64 (27.47%)	
Peer influence				105.34**
With smoking friends	70.4	658 (69.78%)	285 (30.22%)	
Without smoking friends	29.6	379 (95.47%)	18 (4.53%)	

*p < 0.05; **p < 0.01

However, there were two variables not significantly relative with smoking behavior (e.g., domicile place and single-parent family). It seemed that no matter whether people came from countryside or town, the likelihood of occurrences of cigarette smoking did not vary. The phenomenon was the same as whether people belonged to single-parent families or not. The result might support the viewpoint that with the socialization proceeding, the influence power of original family on individual’s behavior and capability would attenuate [36].

### Logistic regression model

Based on the analysis of chi-square test, gender, majors, SES, FE, and peer influence were included in the logistic regression model (LRM). The regression dummy variables in LRM were respectively gender (male = 1, female = 2), majors (pedagogy = 1, medicine = 2, literature and art = 3, engineering = 4, sports = 5, nursing = 6), SES (< 3000 yuan = 1, 3000–10,000 yuan = 2, > 10,000 yuan = 3), FE (singleton family = 1, non-singleton family = 2), peer influence (with smoking friends = 1, without smoking friends = 0).

As for Han was the largest Chinese ethnicity, we renewed the ethnicity variable by encoding Yi, Hani and other minority into one category with Han as the other main category. As shown in Table 2, hypothesis 1 was partially supported by the analytic result that many demographic variables were significantly predictable of smoking behavior except age and ethnicity. That is, just like ethnicity, age has no impact on the likelihood of smoking behavior. It was inconsistent with previous studies probably because of the limited age scope of the involved participants and the unbalanced quantity of each ethnicity in this study.

**Table 2:** Demographic variables in prediction of smoking behavior

Variables	B	S.E	*p*	OR	95% CI
Age	− 0.05	0.08	0.49	0.95	0.81–1.11
Gender (control group = female)	2.26	0.19	0.00	9.55	6.48–14.06
Majors (control group = nursing)					
Pedagogy	0.01	0.37	0.98	1.01	0.49–2.07
Medicine	0.78	0.29	0.00	2.19	1.25–3.85
Literature and art	0.02	0.68	0.97	1.02	0.26–3.86
Engineering	0.16	0.58	0.78	1.17	0.37–3.64
Sports	1.03	0.33	0.00	2.81	0.46–5.41
Ethnicity (control group = minority)					
Han	− 0.01	0.16	0.97	0.99	0.72–1.37
SES (control group ≥ 10,000 yuan)					
< 3000 yuan	− 0.80	0.25	0.00	0.45	0.27–0.73
3000–10,000 yuan	− 0.35	0.23	0.12	0.70	0.44–1.10
FE (control group = non-singleton family)					
Singleton family	− 0.46	0.21	0.03	0.63	0.41–0.96
Peer influence (control group = with smoking friends)					
Without smoking friends	− 1.74	0.27	0.00	0.18	0.10–0.29

Encoding of smoking behavior: 1 = Have ever smoked, 0 = Never smoke

However, the main findings in previous studies were testified to be valid again. College students who were male (OR = 9.55), majoring in medicine and sports (OR_medicine_ = 2.19, OR_sports_ = 2.81), born in non-singleton family (OR = 0.63), with higher family income (OR = 0.45), surrounded with smoking friends (OR = 0.18), were more likely to smoke than female students who were majoring in nursing, born in singleton family with lower family income, surrounded without smoking friends.

Psychological variables including learning burnout and psychological distress seemed not capable of affecting the occurrence of smoking behavior due to the analytic result of logistic model as shown in Table 3. However, learning efficacy, cynicism and emotional exhaustion could significantly influence smoking behavior as were covariant with the total score of learning burnout and psychological distress. This result partially supported hypothesis 2, in which the assumption that all the dimensions of learning burnout could affected smoking behavior as TPB model theoretically implied was proven.

**Table 3:** Psychological variables in prediction of smoking behavior

Factors	B	S.E	*p*	OR	95% CI
Learning efficacy	− 0.02	0.01	0.02	0.98	0.96–0.99
Cynicism	0.06	0.02	0.00	1.06	1.02–1.10
Emotional exhaustion	− 0.05	0.02	0.00	0.95	0.92–0.98
Learning burnout	0.01	0.01	0.10	1.01	0.99–1.02
Psychological distress	− 0.01	0.01	0.77	0.997	0.97–1.02

### Mediation model

According to the analysis above, learning burnout was definitely not able to mediate the relation between psychological distress and smoking behavior. What’s more, psychological distress was also incapable of affecting the inclination of smoking behavior for college students. So in order to delve into the reason for the insignificant path between psychological distress and smoking behavior, we divided the continuous variable psychological distress into four categories by the cut-off points proposed by Wen et al. [42]. That is, students scoring 10–15 were distributed into the least distress group with the best mental health, scoring 16–21 into less distress group, scoring 22–29 into more distress group and scoring 30–50 into most distress group with the worst mental health.

**Fig. 2:**
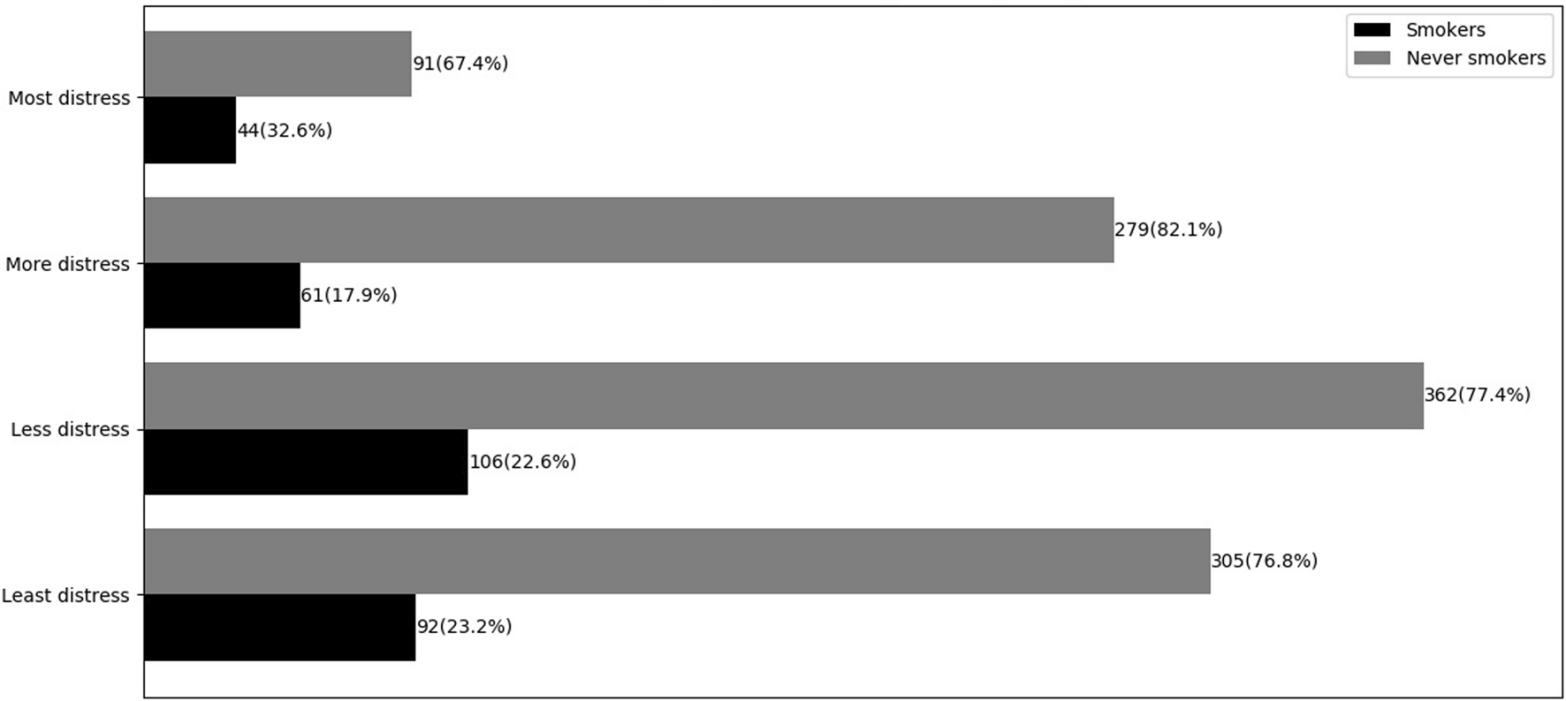
Frequency of different types of psychological distress

As shown in Fig. 2, the descriptive result indicated that college students who fell into most distress group were most likely to smoke (32.6%) with the minimal possibility to become never smokers (67.4%), whereas other groups especially for more distress group were least possible to smoke with the greater possibility to become never smokers on the contrary (e.g., there were 17.89% college students in more distress group belonging to smokers and 82.1% of them belonging to never smokers). The smoking behavior was proven to be correlated with different categories of psychological distress, the chi-square test *χ*^*2*^ between these two variables was 11.99 with statistical significance (*p* < 0.05), but the conclusion that psychological distress could not directly affect smoking behavior remained unchanged.

As mentioned before, psychological distress was correlated with smoking behavior. However, psychological distress could not affect smoking behavior directly. It was possible that there might be a suppressing effect or full mediation effect leading to the insignificant path between psychological distress and smoking behavior. Thus, further analysis to reveal the invisible mechanism behind these two variables is necessary. The mediation method for categorical variable was then carried out.

**Fig. 3:**
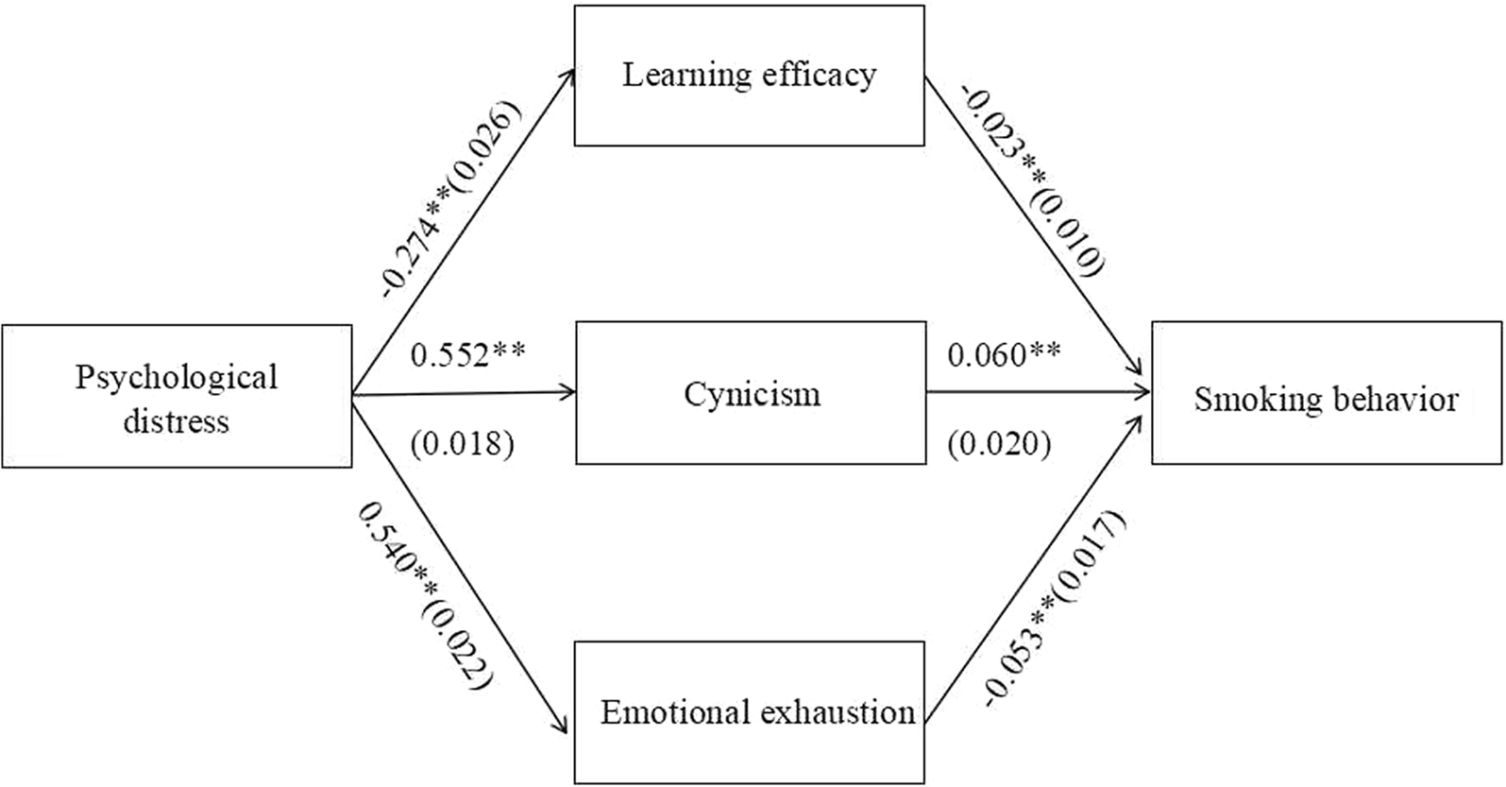
The influence mechanism of psychological distress upon smoking behavior

As shown in Fig. 3, psychological distress can significantly influence smoking behavior only through the dimensions of learning burnout. The *Z* scores for mediation effects of learning efficacy, cynicism and emotional exhaustion were 2.23, 2.98 and − 3.09 respectively, the values of mediation effects all exceeded |1.96| which meant the mediation effects of the dimensions of learning burnout were significant statistically, thus hypothesis 3 was partially supported. A full mediation effect mechanism was revealed in this study that psychological distress could not directly affect the likelihood of smoking behavior, but it could indirectly exert impacts on smoking behavior via learning efficacy, cynicism and emotional exhaustion, the dimensions of learning burnout were found to play an important role on the relationship between psychological distress and smoking behavior.

## Discussion

In the present study, we built up a logistic model including all kinds of demographic variables in the prediction of smoking behavior. It was found that college students who were male, majoring in medicine and sports, born in the non-singleton family, with higher family income, surrounded with smoking friends, were more likely to smoke than female students who were nursing major, born in the singleton family, with lower family income, surrounded without smoking friends. Many findings in previous literature have been testified to be valid in this article. Moreover, we examined the relations between psychological variables and smoking behavior. The intricate relations among these kinds of variables were partially supported. The findings in this article may help the college administrators and college teachers instruct their students to better prevent the tobacco use, it’s also conducive to establish the smoking warning system in which college students who are easy to smoke will be concentrated on.

### The dimensions of learning burnout significantly affect smoking behavior

As for Chinese college students, learning is the main task undertaking great expectations from their parents. Almost every Chinese parents would wish their children have good performances on learning. However, the ones who failed the academic achievement and suffered from learning burnout would undoubtedly bear the enormous stress from outside or inside, as a result, relying on smoking to release the feelings of stress and anxiety was more likely to happen on these students. This study has proven that although learning burnout can not affect smoking behavior due to the broad implication it refers to, its concrete and specific dimensions can significantly affect smoking behavior in somehow.

It has been found in this article that college students with higher learning efficacy and higher emotional exhaustion, in addition to lower cynicism on learning, are hardly to smoke. In explaining this, TPB model can be combined with. Learning efficacy is similar to PBC in the constructs of TPB model, it represents the perceived behavioral control for students. Those students with higher learning efficacy will be more confident of persisting their learning behaviors, their confidence and perseverance grow and the experience can then be transferred to other scenarios such as smoking, they may be more willing and capable of resisting the attempts to smoke especially when they recognize the harmfulness of smoking; Emotional exhaustion as another special construct in learning burnout represents the feeling of tiredness and fatigue after learning. Accompanying with higher emotional exhaustion, students may spend too much time and energy on the daily learning at school, their cognitive resources were exhausted, their energy was run out so that they had no time and enthusiasm to spare on smoking except for a rest, thus higher emotional exhaustion usually indicates less probability of occurrence of smoking behavior; Cynicism mainly implies students’ preferences and perceived social pressure on learning. With higher levels of cynicism, students are more prone to surround by social pressure and exhibit less willingness on learning, thus smoking gradually becomes a visible option for them to relieve the stress, which eventually increase their inclinations to smoke.

### The full mediation effects of dimensions of learning burnout

Although psychological distress can not affect smoking behavior directly, it can indirectly influence smoking behavior through the full mediation mechanism of the dimensions of learning burnout. For example, students with higher psychological distress levels often show lower learning efficacy, and students with lower learning efficacy are easier to become smokers as a result of poor perseverance to inhibit the unhealthy behaviors; Meanwhile, psychological distress can also enhance students’ cynicism toward learning, students immersing in higher levels of psychological distress incline to negatively think of learning and detest learning, therefore they are more vulnerable to smoking culture because smoking has become one of the visible options for them to relieve the great stress caused by the expectations from their families and their inner development motivations. However, it is noteworthy that psychological distress can reversely reduce the likelihood of occurrence of smoking behavior through emotional exhaustion. Students with higher levels of psychological distress are easier to deplete their patience on learning, it will lead them easily to generate negative emotions like sadness, sorrow, anxiety, depression when they keep learning, and it contribute to their increased levels of emotional exhaustion, which may help them resist the attempts to smoke except for a rest.

In conclusion, in order to prevent college students from the tobacco use, the importance of learning should be emphasized. College administrators and teachers can better help their students resist the attempts to smoke indirectly by adjusting college students’ emotions and attitudes toward learning. For example, by lowering the difficulty of curriculum, helping students rebuild their attribution styles and encouraging students to be more confident of their studies, college students’ learning efficacy will be enhanced and their cynicism on learning will be reduced which may contribute to less occurrence of smoking behaviors. Apart from this, college administrators and teachers may also be able to build a better educational environment for college students by attenuating academic competitions and shaping students’ positive learning experiences, students with abundant emotion experiences and strong learning motivations will be more obsessed with the exploration of the ocean of knowledge, more time and energy will be invested on learning for them, as a result, the increased emotional exhaustion will be beneficial for tobacco prevention. In addiction, due to the effects of psychological distress upon learning efficacy and cynicism, college students’ mental health should also be valued. WHO World Mental Health International College Student (WMH-ICS) initiative consisting of epidemiological basis, infrastructure for the development, and the internet intervention for mental health problems could also be adopted for those college students who in the smoking warning system list suffered from psychological distress and mental health problems [46]. Adhering to the mentioned measures and the practical principles, the learning efficacy of college students will be increased as well as their emotional exhaustion, along with the decrease of cynicism, college students can be less willing to smoke in contrast with more probability to concentrate on their school learning.

## Conclusion

This article suggests that although tobacco consumption to some extent enables college students to build up friendships with other people and also relieve their feelings of tension and stress, it would put college students’ physical health in great danger considering the serious addition cigarettes may lead to. Thus, effective tobacco preventive programs and smoking warning system are essential to set up for college students.

In this study, there are 303 college students belonging to smokers accounting for 22.61% of the total sample. College students who were male, majoring in medicine and sports, born in non-singleton family with higher family income, surrounded with smoking friends, were more likely to smoke. The smoking warning system list can be built up based on the demographic features mentioned above, the preventive measures could be taken emphatically to prevent the listed students from tobacco use.

In addition, the article has figured out the specific measures to prevent college students from tobacco use. For instance, due to the full mediation effects of the dimensions of learning burnout, college students’ learning efficacy should be enhanced along with their emotional exhaustion, in addiction to the decreased cynicism on learning, college students will be less willing to smoke.

## Data Availability

The data generated during and/or analyzed during the current study are available from the corresponding author on reasonable request.

## References

[1] Bernhard D (2007). Cigarette smoke–an aging accelerator?. Exp Gerontol.

[2] Vellappally S (2007). Smoking related systemic and oral diseases. Acta Medica.

[3] Watanabe M (2016). Smoking: additional burden on aging and death. Genes Environ.

[4] Barbara E (2013). Smoking-related disease risk, area deprivation and health behaviours. J Public Health.

[5] Jiang Y (2007). Chinese physicians and their smoking knowledge, attitudes, and practices. Am J Prev Med.

[6] Yuan J (2011). Epidemic and control on tobacco in China. Chin J Epidemiol.

[7] Schroergunther M (2011). Primary tobacco prevention in China–a systematic review. Asian Pac J Cancer Prev.

[8] Gruder CL (2012). Tobacco smoking, quitting, and relapsing among adult males in Mainland China: the China Seven Cities study. Nicotine Tob Res.

[9] Lin X, Jie Y (2008). China tobacco related disease and economic burden. Chin J Health Educ.

[10] Johnson CA (2006). Tobacco use among youth and adults in Mainland China: the China Seven Cities study. Public Health.

[11] Shuaijun G (2013). Cluster analysis of smoking, alcohol drinking and other health risk behaviors in undergraduate student. J Peking Univ.

[12] Wang ZG (2009). Comparison of Chinese and Western value from four kinds of angles. J Lianyungang Teach Coll.

[13] Moran S (2004). Social smoking among US college students. Pediatrics.

[14] Rongxian W (2002). Cigarette smoking and tobacco attitudes among undergraduate students in China. J Med Theory Pract.

[15] Hong JJ (2018). Work stress and depressive symptoms in fishermen with a smoking habit: a mediator role of nicotine dependence and possible moderator role of expressive suppression and cognitive reappraisal. Front Psychol.

[16] Picciotto MR (2002). Effect of nicotine and nicotinic receptors on anxiety and depression. NeuroReport.

[17] David GG (1998). Effects of smoking abstinence on mood and craving in men: influences of negative-affect-related personality traits, habitual nicotine intake and repeated measurements. Personal Individ Differ.

[18] Nikcevic AV, Spada MM (2010). Metacognitions about smoking: a preliminary investigation. Clin Psychol Psychother.

[19] Stoliker BE, Lafreniere KD (2015). The influence of perceived stress, loneliness, and learning burnout on university students' educational experience. Coll Stud J.

[20] Maoping L (2015). The correlation of college students’ mental health and learning burnout. J Lishui Univ.

[21] Dan P (2020). Research on coping style improved scores through ameliorating learning burnout. Chin J Health Psychol.

[22] Wei L (2012). Analysis on epidemic status and influencing factors of smoking, drinking and drug addiction among adolescents in Liuzhou. J Med Pest Control.

[23] Yong X, Jing LY (2004). Psychosocial risk factors and family psyche environment of smoking adolescents. Chin Ment Health J.

[24] Wang MQ (2000). Smoking acquisition: peer influence and self-selection. Psychol Rep.

[25] Özer G, Yilmaz E (2011). Comparison of the theory of reasoned action and the theory of planned behavior: an application on accountants' information technology usage. Soc Sci Electron Publ.

[26] Ajzen I (1991). The theory of planned behavior. Organ Behav Hum Decis Process.

[27] Kwan WYM (2009). Predicting physical activity of first-year university students: an application of the theory of planned behavior. J Am Coll Health.

[28] Marc TK (2010). Psychological distress and smoking behavior: the nature of the relation differs by race/ethnicity. Nicotine Tob Res.

[29] Gabriela T, Juan AM (2010). Theory of planned behavior and smoking: meta-analysis and SEM model. Subst Abuse Rehabil.

[30] Mohiyeddini C (2009). The role of emotion in bridging the intention-behaviour gap: the case of sports participation. Psychol Sport Exerc.

[31] Ling L (2014). An investigation about learning burnout in medical college students and its influencing factors. Int J Nurs Sci.

[32] Gentner D (2003). Learning and transfer: a general role for analogical encoding. J Educ Psychol.

[33] Meg F (2017). The association of cigarette smoking with depression and anxiety: a systematic review. Nicotine Tob Res.

[34] Patton GC (1996). Is smoking associated with depression and anxiety in teenagers?. Am J Public Health.

[35] Goodwin RD (2017). Serious psychological distress and smoking during pregnancy in the United States: 2008–2014. Nicotine Tob Res.

[36] Mathias H (2019). Internet interventions for mental health in university students: a systematic review and meta-analysis. Int J Methods Psychiatr Res.

[37] Eisenberg D (2009). Mental health and academic success in college. BE J Econ Anal Policy.

[38] Xiong L (2021). An exploration into the influence of higher vocational students’ psychological capital on their employability. SHS Web Conf.

[39] Qiao H, Schaufeli WB (2009). The factorial validity of the Maslach burnout inventory-student survey in China. Psychol Rep.

[40] Zhang Y (2005). The reliability and validity of MBI-SS and academic characteristics affecting burnout. Chin J Clin Psychol.

[41] Rostami Z (2014). The psychometric characteristics of Maslach burnout inventory student survey: a study students of Isfahan University. Zahedan J Res Med Sci.

[42] Kessler RC (2002). Short screening scales to monitor population prevalences and trends in non-specific psychological distress. Psychol Med.

[43] Xuan W (2015). Mental health and its influence factors among the primary tuberculosis patients. Chin J Health Stat.

[44] Wen GZ (2009). Application of Kessler 10 rating scale in study on relationship between accidental injury and mental health status. Chin Ment Health J.

[45] Iacobucci D (2012). Mediation analysis and categorical variables: the final frontier. J Consum Psychol.

[46] Cuijpers P (2019). Introduction to the special issue: the WHO World Mental Health International College Student (WMH-ICS) initiative. Int J Methods Psychiatr Res.

